# Nomogram to predict 3-month unfavorable outcome after thrombectomy for stroke

**DOI:** 10.1186/s12883-022-02633-1

**Published:** 2022-03-23

**Authors:** Xiao-Guang Zhang, Jia-Hui Wang, Wen-Hao Yang, Xiao-Qiong Zhu, Jie Xue, Zhi-Zhang Li, Yu-Ming Kong, Liang Hu, Shan-Shan Jiang, Xu-Shen Xu, Yun-Hua Yue

**Affiliations:** Department of Neurology, Yangpu Hospital, School of Medicine, Tongji University, 200092 Shanghai, China

**Keywords:** Nomogram, Prediction, Unfavorable outcome, Stroke, Mechanical Thrombectomy

## Abstract

**Background:**

Mechanical thrombectomy (MT) is an effective treatment for large-vessel occlusion in acute ischemic stroke, however, only some revascularized patients have a good prognosis. For stroke patients undergoing MT, predicting the risk of unfavorable outcomes and adjusting the treatment strategies accordingly can greatly improve prognosis. Therefore, we aimed to develop and validate a nomogram that can predict 3-month unfavorable outcomes for individual stroke patient treated with MT.

**Methods:**

We analyzed 258 patients with acute ischemic stroke who underwent MT from January 2018 to February 2021. The primary outcome was a 3-month unfavorable outcome, assessed using the modified Rankin Scale (mRS), 3–6. A nomogram was generated based on a multivariable logistic model. We used the area under the receiver-operating characteristic curve to evaluate the discriminative performance and used the calibration curve and Spiegelhalter’s Z-test to assess the calibration performance of the risk prediction model.

**Results:**

In our visual nomogram, gender (odds ratio [OR], 3.40; 95%CI, 1.54–7.54), collateral circulation (OR, 0.46; 95%CI, 0.28–0.76), postoperative mTICI (OR, 0.06; 95%CI, 0.01–0.50), stroke-associated pneumonia (OR, 5.76; 95%CI, 2.79–11.87), preoperative Na (OR, 0.82; 95%CI, 0.72–0.92) and creatinine (OR, 1.02; 95%CI, 1.01–1.03) remained independent predictors of 3-month unfavorable outcomes in stroke patients treated with MT. The area under the nomogram curve was 0.8791 with good calibration performance (*P* = 0.873 for the Spiegelhalter’s Z-test).

**Conclusions:**

A novel nomogram consisting of gender, collateral circulation, postoperative mTICI, stroke-associated pneumonia, preoperative Na and creatinine can predict the 3-month unfavorable outcomes in stroke patients treated with MT.

**Supplementary Information:**

The online version contains supplementary material available at 10.1186/s12883-022-02633-1.

## Background

Acute ischemic stroke (AIS) is a common cerebrovascular event that can easily cause disability [1]. Mechanical thrombectomy (MT) is essential for AIS with large-vessel occlusion, of which blood perfusion can rarely be restored by intravenous thrombolysis [2]. With the popularization of MT, more and more patients have successfully achieved recanalization. However, a successful recanalization of the occluded artery does not always lead to a good outcome (90-day modified Rankin Scale ≥ 2) [3]. Therefore, it is especially important to explore other potential clinical factors that affect the prognosis of thrombectomy.

In the recent years, several scores based on clinical and radiological variables have been used to predict the 3-month outcomes of acute anterior circulation stroke after MT [4, 5], such as the GADIS (Gender, Age, Diabetes Mellitus History, Infarct Volume, and Current Smoker) score and MT-DRAGON (adapt the “MRI-DRAGON” for mechanical thrombectomy) score. However, these scores still have many shortcomings, the most important of which is that the performance for individualized prediction of outcome is limited by the use of categorization of continuous variables, such as age, glucose level, ASPECT (Alberta Stroke Program Early CT) score, and National Institutes of Health Stroke Scale (NIHSS) score. Nomogram is a computational tool that can generate numerical probabilities of clinical events based on each patient’s personal profile by creating graphical representation of statistical prediction models [6], which has demonstrated significant advantages over traditional staging systems and has been used to predict many diseases, such as pneumonia, myocardial infarction, and cancer [7–9]. Based on its advantage to use continuous variables, nomogram is a better visual tool than the above scores to predict the 3-month outcomes after MT.

Recently, several nomograms have been constructed for predicting 3-month outcome after thrombectomy for stroke [3, 10, 11]. But the patients included in the studies mainly had symptoms of acute anterior circulation stroke within 6 h of onset. With the update of the guideline about endovascular thrombectomy, especially the extension of the time windows and the generation of new devices, more patients with indications are suitable for thrombectomy [12]. Therefore, new nomograms for AIS patients undergoing MT are urgently needed.

Given that 3-month is a fairly representative timepoint of the long-term outcomes of MT for stroke [13], we selected 3-month as the prognostic observation point of MT. The present study aimed to develop and validate a nomogram to predict 3-month unfavorable functional outcomes in Chinese AIS patients undergoing MT by using a limited number of easily available variables.

## Methods

### Study population

We conducted a retrospective longitudinal study based on data collected from January 2018 to February 2021 from AIS patients who were treated by MT therapy in the Department of Stroke of the Yangpu Hospital, Tongji University School of Medicine. Participants in this study was reviewed and approved by ethics committee of Yangpu Hospital (ethical approval number LL-2018-SCI-004). Informed consent has been obtained. Patients were included in this study if all the following conditions were met: (1) aged ≥ 18 years; (2) pre-stroke modified Rankin Scale score (mRS) of 0 to 1; (3) with occlusion of the internal carotid artery, anterior cerebral artery, middle cerebral artery, or vertebrobasilar artery confirmed by digital subtraction angiography; (4) undergoing MT treatment within 24 h of the stroke onset. Patients with the onset for more than 6 h further received the evaluation of CT scan perfusion and thrombectomy was performed among those who meet the DAWN or DEFUSE 3 eligibility criteria [14, 15], and patients will receive antithrombotic drugs following the MT treatment. Patients with haemorrhage on baseline or within 24 h after MT, lost to follow-up without 3-month mRS score, baseline or laboratory data and pre-/post-operative imaging examination were unavailable were excluded.

### Data collection

Demographic characteristics, vascular risk factors, clinical data, radiological images, and laboratory data were all collected. These include age, gender, baseline NIHSS score, baseline ASPECTS score, thrombolysis time window, thrombolysis, anesthesia, vascular risk factors such as hypertension, diabetes mellitus, coronary heart disease, atrial fibrillation, stroke, smoking and alcoholism, clinical data such as systolic/ diastolic blood pressure, time from onset to puncture, time from puncture to recanalization, bridging therapy (combined treatment with intravenous alteplase (at a dose of 0.9 mg per kilogram of body weight) before MT), number of retrieval attempts, and stroke-associated pneumonia (SAP), radiological images such as TOAST classification, collateral circulation, vascular occlusion site, postoperative modified Thrombolysis in Cerebral Infraction (mTICI) score and symptomatic intracranial hemorrhage (sICH), preoperative laboratory data such as C-reactive protein(CRP), red blood cell distribution width (RDW), hemoglobin (HGB), glucose, Na concentration, creatinine and B-natriuretic peptide (BNP).

The primary outcome was the unfavorable functional consequences at 3 months after MT for stroke, with mRS between 3 and 6 (i.e., poor prognosis). The 3-month mRS were evaluated by trained physicians with telephone questionnaires or face-to-face interviews.

### Statistical analysis

Continue variables were described as median (interquartile intervals 25–75), and univariate comparisons were performed using Mann–Whitney U where appropriate. Categorical variables were expressed as frequencies and percentages, and differences were assessed by the chi-square test or Fisher’s exact test. The glmnet package in R was used for the least absolute shrinkage and selection operator (LASSO) logistic regression, and significant factors there in were used to enter into the logistic regression model. Using forward stepwise selection with Akaike’s information criterion (AIC) as the stopping rule, variables included in the final multivariable logistic regression model were carefully chosen. The AIC value for the final model was minimized with the fewest number of variables. The Variation Inflation Factors (VIF, < 10 indicating no multicollinearity problem) was used to evaluate the collinearity of combination variables entered into the multivariable logistic regression analysis. The odds ratio (OR) and 95% confidence interval (95% CI) were calculated for variables significantly associated with the main outcome in the multivariable analysis. Factors remained significant in the multivariable analysis were incorporated in the nomogram.

Based on discrimination and calibration, the discriminative power of the model was assessed. The discrimination of the nomogram model was validated using the receiver operating characteristic (ROC) curve and quantified by the area under the curve (AUC), while the calibration was visually assessed using the calibration plot and the Spiegelhalter’s Z-test. A 45° diagonal line indicates perfect calibration when the predictive value of a well-calibrated model perfectly matches the patient’s actual risk. Nomogram construction and calibration was assessed graphically by means of the R package rms.

The statistical analysis was carried out using Stata version 15.0 (Stata Corporation, College Station, TX, USA) statistical software and the statistical software package R version 3.6.0. All tests were two-sided and *P* < 0.05 was considered statistically significant.

## Results

Two hundred and fifty-eight AIS patients were finally enrolled in the entire study (mean age, 72.90 ± 12.41 years), 219 patients were treated within 6 h of symptom onset (Time from onset to groin puncture) and 39 patients treated between 6 and 24 h after onset of symptom onset. One hundred and twenty-nine patients received bridging therapy. The proportion of patients with poor 3-month pronosis (mRS score 3–6) was 58.9% (152/258) and 22.87% (59/258) of patients died during the follow-up period (mRS score = 6). Among them, 43 died of vascular death, 2 died of recurrent ischemic stroke, and 14 died of Non-vascular death (Table 1).

**Table 1:** Stratification of demographics and clinical characteristics of the study population based on favorable or unfavorable outcome at 3 months in Chinese patients with acute ischemic stroke treated with mechanical thrombectomy

Variable	Favorable outcome (mRS 0–2)	Unfavorable outcome (mRS 3–6)	*P* Value
Patients, n (%)	106	152	
Demographic characteristics			
Age, y	68.97 ± 12.55	75.63 ± 11.59	< 0.001
Female, n (%)	33(31.13)	88(57.89)	< 0.001
Vascular risk factors, n (%)			
Hypertension	64(60.38)	109(71.71)	0.057
Diabetes mellitus	20(18.87)	49(32.24)	0.017
Atrial fibrillation	28(26.42)	55(36.18)	0.098
Coronary heart disease	22(20.75)	42(27.63)	0.208
Stroke	20(18.87)	40(26.32)	0.164
Smoking	51(48.11)	49(32.24)	0.010
Alcoholism	7(6.60)	7(4.61)	0.486
Clinical data			
Systolic blood pressure, mm Hg	152.35 ± 20.99	152.49 ± 25.82	0.962
Diastolic blood pressure, mm Hg	85.64 ± 15.91	85.70 ± 14.64	0.977
Baseline NIHSS, score	13.21 ± 6.85	16.28 ± 6.81	< 0.001
Baseline ASPECTS, score	8(7–9)	7(6–8.75)	0.039
Thrombolysis time window	74(69.81)	82(53.95)	0.010
Thrombolysis	65(61.32)	64(42.11)	0.002
Time from onset to thrombolysis, min	106(79–135.5)	123.5(90–189)	0.024
Mechanical thrombectomy > 6 h, n (%)	13(12.26)	26(17.11)	0.286
Time from onset to puncture, min	200.00(135.00–273.75)	222.50(160.00–332.50)	0.042
Time from puncture to recanalization, min	90(55.25–135.00)	87.5(50.00–160.00)	0.450
General Anesthesia	29(27.36)	41(26.97)	0.945
Bridging therapy	65(61.32)	64(42.11)	0.002
More than one Retrieval Attempt	43(40.57)	87(57.24)	0.008
TOAST classification, n (%)			
Large artery atherosclerosis	63 (59.43)	76(50.00)	0.307
Cardioembolism	41(38.68)	72(47.37)	
Others	2(1.89)	4(2.63)	
Collateral circulation, n (%)			
	2(1.89)	19(12.50)	< 0.001
	13(12.26)	37(24.34)	
	67(63.21)	83(54.61)	
	24(22.64)	13(8.55)	
Vascular occlusion site, n (%)			
ICA	26(24.53)	59(38.82)	0.010
MCA	69(65.09)	70(46.05)	
VA	11(10.38)	23(15.13)	
Postoperative mTICI (≥ 2b)	105(99.06)	128(84.21)	< 0.001
Stroke-associated pneumonia	29(27.36)	108(71.05)	< 0.001
sICH, n (%)	12(11.32)	30(19.74)	0.072
Laboratory data			
CRP(mmol/l)	5.0(2.85–5.00)	5.14(3.10–13.31)	0.003
RDW(%)	12.95 (12.50–13.40)	13.00 (12.60–13.93)	0.096
HGB(g/l)	142.05 ± 19.37	131.86 ± 20.58	< 0.001
GLU(mmol/l)	6.71(6.01–8.09)	8.35(6.70–10.21)	< 0.001
Na(mmol/L)	140.15 ± 2.68	139.18 ± 3.32	0.014
Creatinine (μmol/L)	68.65 (60.0–82.75)	74.00 (61.00–92.00)	0.064
BNP(ng/l)	107.00 (22.25–270.50)	234.00 (98.00–493.25)	< 0.001
Death			NA
Vascular death	0	43	
Recurrent ischemic stroke	0	2	
Non-vascular death	0	14	

The clinical, demographic and laboratory characteristics of the patients in the favorable outcome cohorts (*n* = 106) and unfavorable outcome (*n* = 152) cohorts are shown in Table 1. The differences between patients with unfavorable outcome and favorable outcome in age (*p* < 0.001), gender (*p* < 0.001), diabetes mellitus (*p* = 0.017), smoking (*p* = 0.010), baseline NIHSS (*p* < 0.001), baseline ASPECTS (*p* = 0.039), thrombolysis time window (*p* = 0.010), thrombolysis (*p* = 0.002), time from onset to puncture (*p* = 0.042), bridging therapy (*p* = 0.002), more than one retrieval attempt (*p* = 0.008), collateral circulation (*p* < 0.001), vascular occlusion site (*p* = 0.010), postoperative mTICI (*p* < 0.001), SAP (*p* < 0.001), CRP (*p* = 0.003), HGB (*p* < 0.001), GLU (*p* < 0.001), Na (*p* = 0.014) and BNP (*p* < 0.001) are significant.

**Fig. 1:**
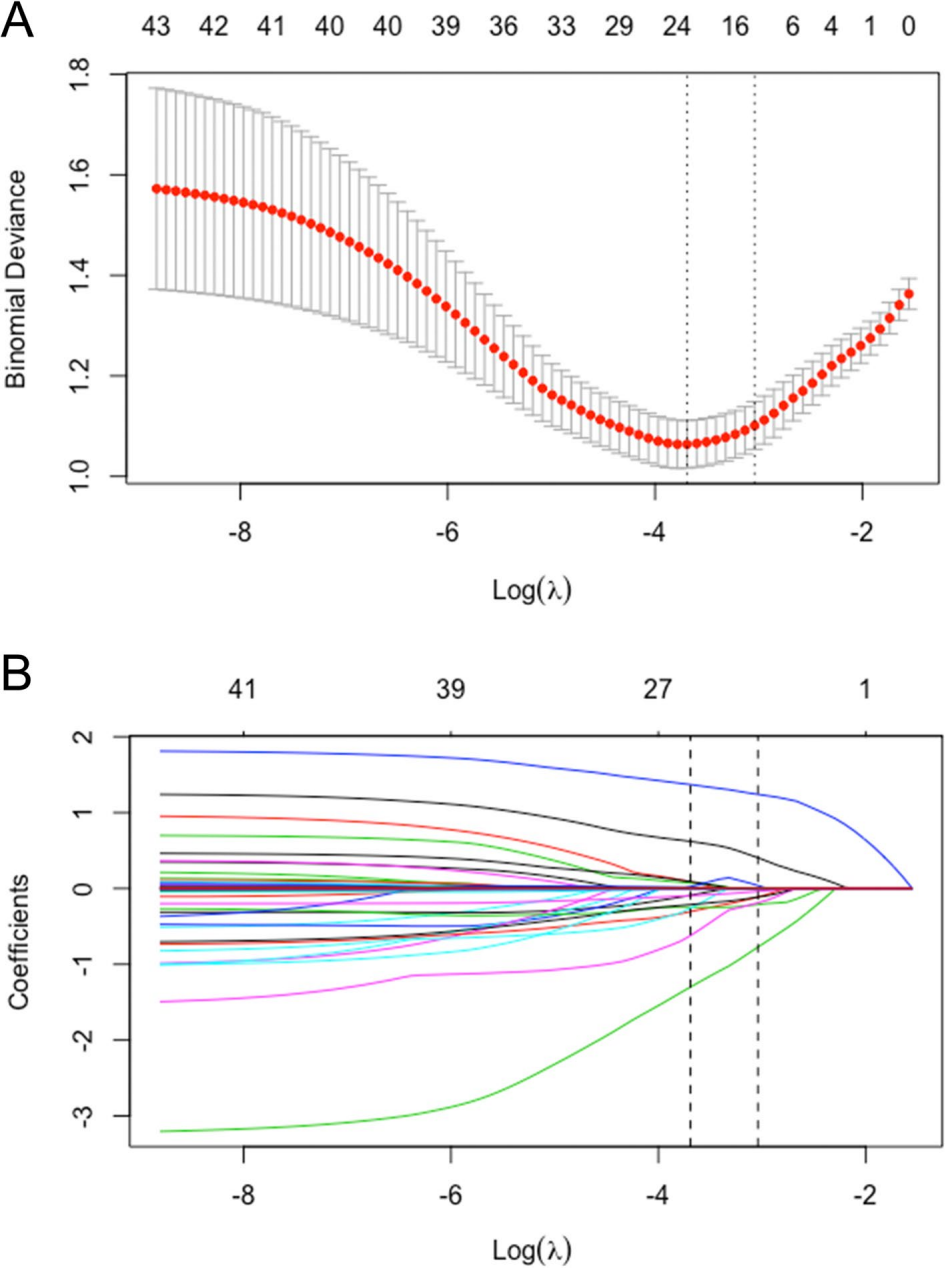
Factor selection using the least absolute shrinkage and selection operator (LASSO) logistic regression. **A** The LASSO coefficient profiles of the 40 candidate variables. The binomial deviance is plotted versus log (λ). **B** Tuning parameter (λ) selection in the LASSO logistic regression performed using tenfold cross-validation via the minimum criteria. A coefficient profile plot is produced versus the log (λ). The left vertical line represents the minimum error, and the right vertical line represents the cross-validated error within 1 standard error of the minimum

The LASSO logistic regression algorithm were used to select the most significant predictive factors, which were then used to construct the prediction model. A total of 40 factors showed in Table 1 and S1 were used for the LASSO logistic regression, and 13 factors with non-zero coefficients were subsequently selected, with an optimal lambda value of 0.025 (Fig. 1A, B). The model ultimately included 13 factors: age, gender, baseline NIHSS, baseline ASPECTS, thrombolysis time window, thrombolysis, bridging therapy, collateral circulation, vascular occlusion site, postoperative mTICI, SAP, preoperative HGB, Na, and creatinine.

**Fig. 2:**
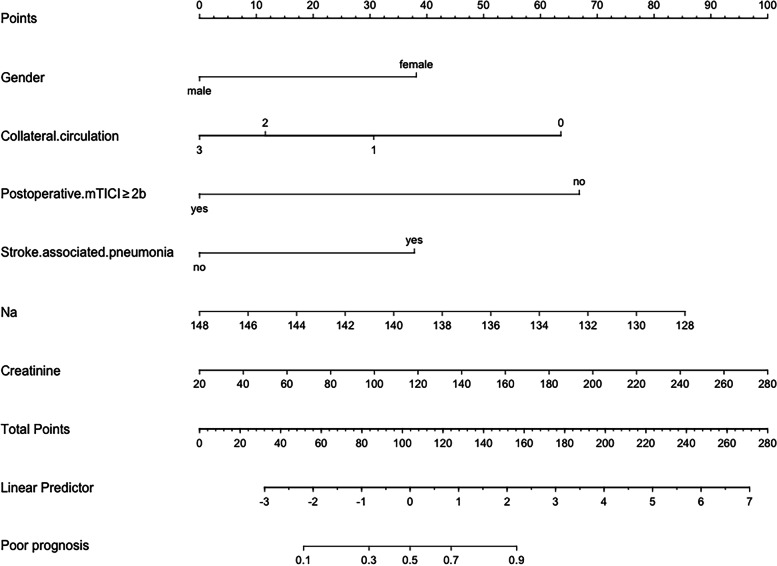
Nomogram for predicting the probability of 3-month unfavorable outcome in acute ischemic stroke patients undergoing mechanical thrombectomy based on gender, collateral circulation, postoperative mTICI, stroke-associated pneumonia, preoperative Na and creatinine

After multivariable logistic regression analysis, gender (OR: 3.40, *p* = 0.003), collateral circulation (OR: 0.46, *p* = 0.002), postoperative mTICI (OR: 0.06, *p* = 0.010), SAP (OR: 5.76, *p* < 0.001), preoperative Na (OR: 0.82, *p* = 0.001) and creatinine (OR: 1.02, *p* = 0.011) remained independent predictors of 3-month unfavorable outcome (Table 2, Fig. 2). No significant statistical collinearity was observed for any of these six variables. The logistic regression model was generated as: Log [p(x)/1-p(x)] = 31.77 + (1.22 * gender)—(0.77 * collateral circulation)—(2.90 * postoperative mTICI) + (1.75 * SAP)—(0.20 * preoperative Na) + (0.02 * preoperative creatinine).

**Table 2:** Multivariable logistic regression model for adverse prognostic indicators in Chinese patients with acute ischemic stroke undergoing mechanical thrombectomy

	β	SE	Odds Ratio (95% CI)	*P* value
Gender	1.22	0.41	3.40 (1.54 to 7.54)	0.003
Collateral circulation	-0.77	0.25	0.46 (0.28 to 0.76)	0.002
Postoperative mTICI	-2.90	1.12	0.06 (0.01 to 0.50)	0.010
Stroke-associated pneumonia	1.75	0.37	5.76 (2.79 to 11.87)	< 0.001
Na	-0.20	0.06	0.82 (0.72 to 0.92)	0.001
Creatinine	0.02	0.01	1.02 (1.01 to 1.03)	0.011

**Fig. 3:**
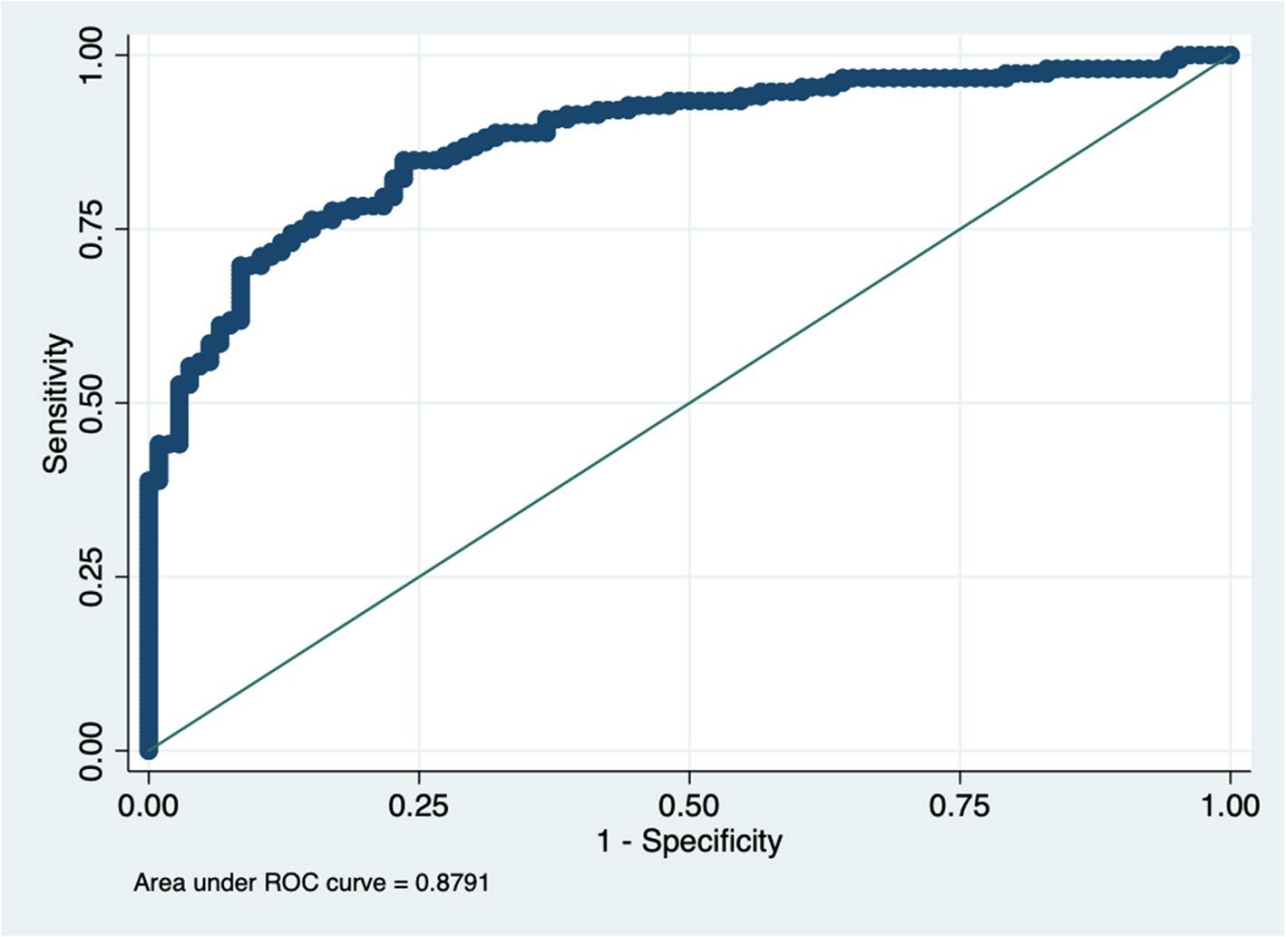
Receiver operating characteristic (ROC) curve of the nomogram for predicting 3-month unfavorable outcomes of stroke patients treated with mechanical thrombectomy

**Fig. 4:**
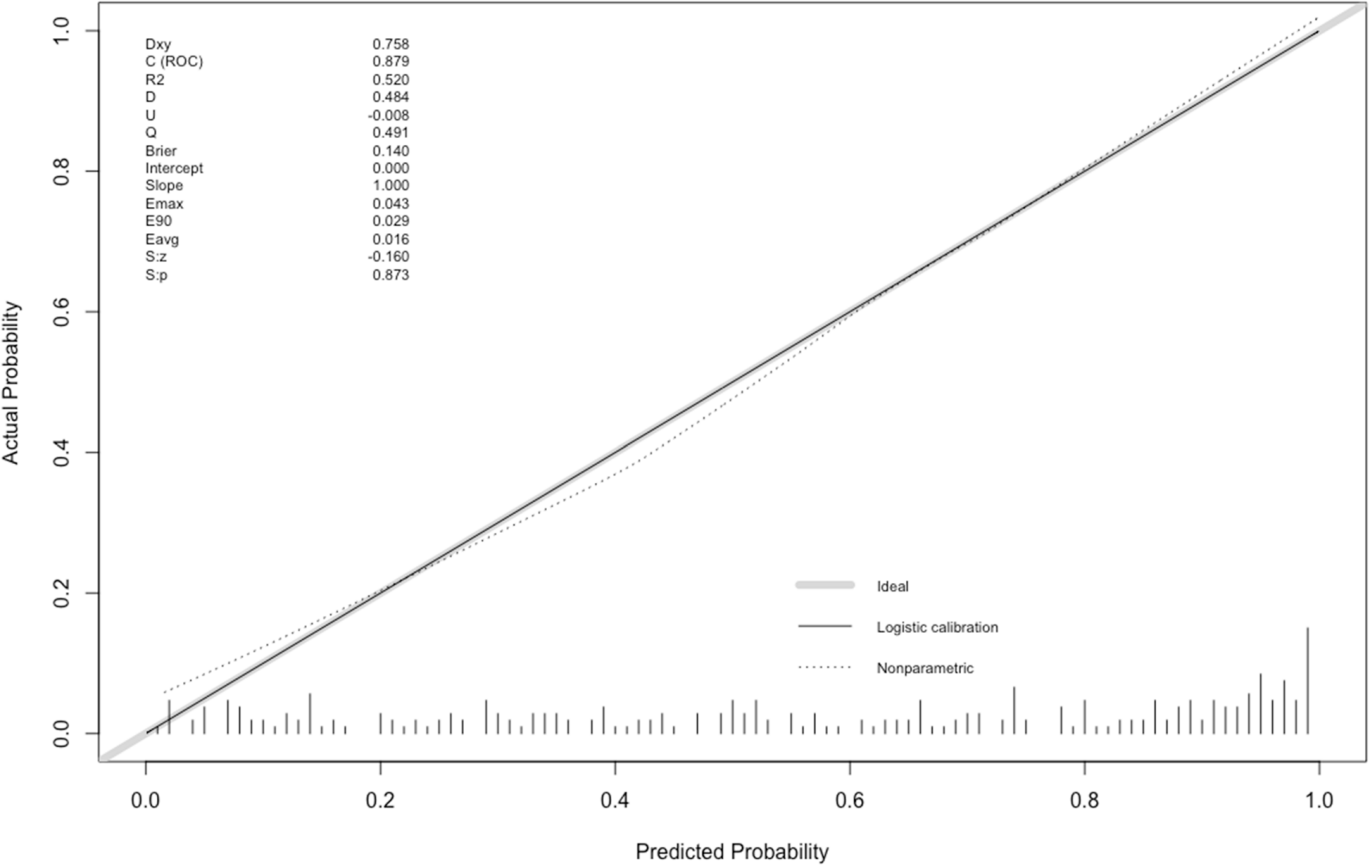
The calibration plot of the nomogram for predicting 3-month unfavorable outcomes of stroke patients treated with mechanical thrombectomy. The x-axis represents the predicted probability of unfavorable outcome calculated using the nomogram. The y-axis represents the actual rate of unfavorable outcome. The dashed line is the reference line where an ideal nomogram would lie. The dotted line is the performance of the nomogram, while the solid line corrects for any deviation of the nomogram

A nomogram was created by assigning a graphical preliminary score ranging from 0 to 100 to each of the six predictors, then summing them to generate a total score, which was then converted into an individual probability of 3-month unfavorable outcome (from 10 to 90%) (Fig. 2). It was predicted that a higher total score of the nomogram was associated with a higher likelihood of unfavorable outcome, while a lower total score was associated with a lower likelihood of an adverse outcome. In this cohort, the nomogram had an area under the curve (AUC) of 0.8791 (95% CI: 0.84–0.92) (Fig. 3). The *p*-value for Spiegelhalter’s Z-test was 0.873, which indicated that there was no significant departure from a perfect fit. The Brier was 0.140, which meant that the calibration curve was almost close to the ideal curve. The calibration plot revealed the adequate fit of the model, predicting the risk of the poor prognosis at 3 months after MT (Fig. 4).

## Discussion

### Major findings

For precise clinical and therapeutic management of AIS patients, early prediction of unfavorable outcome is undoubtedly a valuable perspective. In this study, we developed a nomogram model to predict the unfavorable outcome after MT in AIS with large-vessel occlusion. Our nomogram provides a quantitative prediction of a 10% to 90% probability of 3-month unfavorable outcome in Chinese AIS patients undergoing MT, and it exhibits an excellent predictive power with a C-index of 0.8791. Gender, collateral circulation, postoperative mTICI, SAP, preoperative Na and creatinine are independently associated with poor prognosis at 3-months.

Although 90.31% of the patients (233/258) benefited from the progressive development of MT intervention materials and prolonged treatment time window to achieve postoperative mTICI (≥ 2b), only 45.49% of the patients (106/233) benefited from the reperfusion. At present, only limited data are available for clinicians to determine the risk of poor prognosis after MT in AIS with large-vessel occlusion. Due to the paucity of data, this nomogram was developed specifically for patients undergoing MT within 24 h from onset to treatment since 2018. This nomogram is based on six basic clinical data, three preoperative data (gender, creatinine and Na), one intraoperative data (collateral circulation), and two postoperative data (mTICI, SAP), which demonstrated an excellent discrimination in terms of internal validation.

### Nomogram variables interpretation

In our study, by using a combination of six easily available predictors before and after endovascular procedure, the area under the curve (AUC) of our nomogram could reach 0.8791, which means it may be a novel and reliable graphical computational tool that can provide guidance for AIS patient management after revascularization treatments. Gender differences is also one of the factors affecting the prognosis of stroke. Previous studies reported that females consistently fare worse than males following MT for large vessel ischemic stroke [16, 17]. One of the possible reasons is that males have larger cerebral arterial diameters than females, which may improve the odds for favorable clinical outcomes [18]. Serum creatinine is widely used as an indicator of renal function, and impaired renal function is also associated with long-term mortality and poor prognosis after stroke [19]. The present study is consistent with previous findings that the worse the renal function, the worse the prognosis. Sodium ions play an important role in maintaining intracellular and extracellular electrolyte gradients, nerve conduction velocity, and muscle excitability [20]. Mild hyponatremia is known to be an independent prognostic factor for mortality in AIS patients [21]. Although the average preoperative Na concentration in the poor prognosis group has not reached hyponatremia, but was lower than that in the good prognosis group, indicating that it could affect the prognosis to some extent. The lower the preoperative Na concentration, the greater the risk of unfavorable outcomes. The collateral circulation of the brain consists of arterial anastomotic pathways capable of supplying nutrient perfusion to brain regions whose primary source of blood flow have become compromised by acute ischemic stroke [22]. Collateral circulation is a key determinant of functional outcome after MT after large-vessel ischemic stroke [23]. Our study also concluded that the better the collateral circulation, the better the functional outcome. Successful recanalization after MT is the strongest adjustable predictor of patient prognosis, and higher recanalization rates are associated with functional improvement at 3 months [24]. Successful recanalization of large-vessel occlusions is traditionally defined by mTICI grades of 2b or 3. Our study also confirmed that the patient prognosis of mTICI greater than 2b was better. Moreover, SAP is another strong predictor of poor outcome in AIS patients undergoing MT. Aspiration caused by dysphagia, reduced consciousness, impaired bulbar reflexes, and hypokinesia are more likely to cause poststroke cumulus pneumonia in AIS patients with MT [25]. For patients with acute stroke, the occurrence of pneumonia has been shown to be associated with poor functional-reelated outcomes and increased risk of death [26]. For patients with acute ischemic stroke due to anterior circulation large-vessel occlusion, they are eligible for both intravenous alteplase and MT according to the update of guidelines. The Direct Intraarterial Thrombectomy in Order to Revascularize Acute Ischemic Stroke Patients with Large Vessel Occlusion Efficiently in Chinese Tertiary Hospitals: a Multicenter Randomized Clinical Trial (DIRECT-MT) have shown that direct intervention with MT was noninferior to the combination of intravenous thrombolysis followed by MT [27]. In the present study, we also found that bridging therapy was not an independent predictor of 3-month unfavorable outcome. Due to the small sample size of our study, this result should also be interpreted with caution, and further analysis of other large research is therefore warranted. Therefore, our nomogram covers preoperative, intraoperative and postoperative variables and may be more practical in some respects.

### Comparison with prior studies

Previously, several models have been developed to predict the probability of 3-month mortality in AIS patients receiving MT [3, 10, 11]. Among them, an Italian cohort study developed a nomogram that included NIHSS score, age, pre-stroke mRS score, bridging therapy, direct thrombectomy, grade of recanalization according to the mTICI grading system, and onset-to-end procedure time [10]. While two Chinese studies identified many demographic and clinical characteristics, including baseline NIHSS score, age, creatinine, blood glucose level, collateral status, and sICH [3, 11]. Due to the inconsistent inclusion and exclusion criteria, the potential predictors finally included in their nomograms were quite different. The patients in Cappellari’s study had anterior and posterior large-vessel occlusion who underwent thrombectomy within 6 h of stroke onset [10]. With the update of the guidelines on thrombectomy, the time window for thrombectomy has been expanded from 6 to 24 h for patients with large-vessel occlusion in the anterior circulation and meeting DAWN or DEFUSE 3 eligibility criteria [12], and the number of patients requiring endovascular treatment has increased accordingly. Therefore, it is currently necessary to update the nomogram to predict the probability of unfavorable outcome in AIS patients who are candidates for thrombectomy. Zhang et al. developed a nomogram that predicted the likelihood of 3-month mortality in patients with anterior circulation stroke who had a successful EVT within 6 h of symptom onset [3]. Patients with unsuccessful recanalization, non-anterior circulation stroke, exceeding 6 h of last known normal values were excluded from their study. Based on the fact that unsuccessful recanalization is often accompanied with poor functional outcomes, many patients with poor prognosis may be excluded. While our study assessed AIS patients undergoing MT treatment within 24 h of the stroke onset with both anterior and posterior circulation infarction.

### Nomogram clinical consideration

We could easily obtain the most significant variables in the nomogram for each patient, indicating that this nomogram is highly practical as a predictive tool. Unlike previous prognostic models [28, 29], our nomogram assigns a probability (from 10 to 90%) of unfavorable outcome. For example, our nomogram assigns > 90% probability of adverse consequence when a Female patient (38 point), with level 2 collateral circulation (12 points), postoperative mTICI < 2b is 67 points, SAP is 37.5 points, preoperative Na concentration is 134 mmol/l (60 points) and creatinine concentration is 120 mmol/l (38 points), with a total score of 252.5 points. On the other hand, a male patient (0 point), with level 3 collateral circulation (0 points), postoperative mTICI ≥ 2b (0 point), no SAP (0 points), preoperative Na concentration is 144 mmol/l (17 points) and creatinine concentration is 60 mmol/l (15 points), with a total score of 32 points, produce a probability of adverse outcome of < 10%.

By converting the total score into a continuum of individual probabilities, our nomogram can more precisely reclassify the risk of 3-month adverse outcome. Thus, the nomogram may better identify different risk predictors in AIS patients undergoing MT than previous prognostic models based on inadequate risk-grouping categorization [30].

### Limitation

There are several limitations in this study. First, the data we collected were from a single center, so the sample size was not large enough, which might limit the generalizability of our findings. Further expansion of the sample size is needed to improve the nomogram. Second, biomarkers, such as brain edema and infarct size, were not included in the model because these parameters were not available in the current imaging system. Our future prospective study will include more parameters and determine whether the integration of other biomarkers may help to improve the accuracy of the nomogram. Third, this study is based on a retrospective analysis of prospectively collected data. Despite the good discriminative performance of this model, external validation in completely different patient cohorts is still required.

## Conclusions

A novel nomogram consisting of gender, collateral circulation, postoperative mTICI, SAP, preoperative Na and creatinine can better predict the 3-month unfavorable outcomes in stroke patients treated with MT.

## Supplementary Information

**Additional file 1: Table S1.** Supplementary Demographics and Clinical Characteristics of study population stratified according to 3-month favorable or unfavorable outcome after acute ischemic stroke in Chinese patients with mechanical thrombectomy.

## Data Availability

The original contributions presented in the study are included in the article, further inquiries can be directed to the corresponding author.

## Abbreviations

MT: Mechanical thrombectomy
NIHSS: National Institutes of Health Stroke Scale
ASPECTS: Alberta Stroke program early CT score
TOAST: Trial of Org 10,172 in Acute Stroke Treatment
ICA: Internal carotid artery
MCA: Middle cerebral artery
VA: Vertebrobasilar artery
mTICI: Modified Thrombolysis in Cerebral Infraction
SAP: Stroke-associated pneumonia
sICH: Symptomatic intracranial hemorrhage
CRP: C-reactive protein
RDW: Red blood cell distribution width
HGB: Hemoglobin
GLU: Glucose
BNP: B-natriuretic peptide
N/A: Not applicable
