# Supplementary material for: Nomogram to predict 3-month unfavorable outcome after thrombectomy for stroke
Source: BMC Neurol. 2022 Mar 23;22:111. doi: 10.1186/s12883-022-02633-1 (PMC8941794; doi:10.1186/s12883-022-02633-1)
Supplement: Supplementary file 1 — Additional file 1: Table S1. Supplementary Demographics and Clinical Characteristics of study population stratified according to 3-month favorable or unfavorable outcome after acute ischemic stroke in Chinese patients with mechanical thrombectomy. [file 12883_2022_2633_MOESM1_ESM.docx]

Table S1. Supplementary Demographics and Clinical Characteristics of study population stratified according to 3-month favorable or unfavorable outcome after acute ischemic stroke in Chinese patients with mechanical thrombectomy.

| Variable | Favorable outcome  (mRS 0–2) | Unfavorable outcome  (mRS 3–6) | P Value |
| --- | --- | --- | --- |
| Patients, n | 106 | 152 |  |
| Previous medication |  |  |  |
| Antiplatelet drugs, n (%) | 20(18.87) | 38(25.00) | 0.246 |
| Anticoagulant, n (%) | 10(9.43) | 12(7.89) | 0.663 |
| Antihypertensive drugs, n (%) | 64(60.38) | 93(61.18) | 0.896 |
| Hypoglycemic drugs, n (%) | 20(18.87) | 38(25.00) | 0.246 |
| Statin, n (%) | 17(16.04) | 28(18.42) | 0.620 |
| Thrombolytic contraindication |  |  |  |
| Recent cerebral infarction, n | 1 | 1 |  |
| Anticoagulants, n | 6 | 8 |  |
| Scalp hematoma, n | 1 | 0 |  |
| Previous cerebral hemorrhage, n | 2 | 2 |  |
| Low density changes in early infarction, n | 1 | 1 |  |
| Reject, n | 1 | 1 |  |
| Aplastic anemia, n | 0 | 1 |  |
| Thrombocytopenia, n | 0 | 1 |  |
| Previous surgery, n | 0 | 4 |  |
| Abnormal coagulation, n | 0 | 1 |  |
| Other infections |  |  |  |
| Urinary tract infection | 2 | 1 |  |
| Sepsis | 0 | 1 |  |
| Cholecystitis | 0 | 1 |  |
| Bacteremia | 1 | 0 |  |
